# Effect of red yeast rice on the growth of male SD rats: a 90-day feeding study

**DOI:** 10.1007/s43188-025-00297-w

**Published:** 2025-05-20

**Authors:** Hayoung Lee, Byungkyung Do, Hoonjeong Kwon

**Affiliations:** 1 Department of Food and Nutrition, Seoul National University, Gwanak-ro 1 Gwanak-gu, Seoul, 08826 Republic of Korea; 2 College of Pharmacy, Chungnam National University, Daejeon, 34134 Republic of Korea; 3 Research Institute of Human Ecology, Seoul National University, Seoul, 08826 Republic of Korea

**Keywords:** Red yeast rice, 90-day feeding study, SD rats, Growth inhibition

## Abstract

Red yeast rice (RYR) is commonly used as colouring and flavouring agent in foods throughout East Asia. RYR contains monacolin K, a compound known to lower blood lipids level, which has led to its use as a health functional food in Korea. Additionally, RYR is frequently used as a food ingredient and is incorporated into processed foods such as bread and makgeolli, a traditional Korean alcoholic beverage. However adverse effects associated with RYR have been reported by several regulatory agencies, prompting the need for further investigation of its safety as a general food ingredient. To evaluate its safety, a 90-day feeding study was conducted using Sprague–Dawley (SD) rats, which were randomly assigned to three experimental groups. The experimental diets were prepared by replacing the corn starch in AIN 93G with RYR and/or white rice. Rats fed RYR showed lower body weight gain, accompanied by reduced food efficiency. No signs of toxicity were observed in serum clinical chemistry, relative organ weights, or histopathological analysis. However, hyperplasia and hypertrophy were observed in the thyroid, although the cause remained unclear. These results suggest that RYR exhibits a very low toxic potential, if any. Nevertheless, caution is advised regarding its expanded use, particularly among younger population, due to its growing inhibitory effects.

## Introduction

Red yeast rice (RYR) is a fermented product of ordinary rice (*Oryza sativa*) with certain mold species of *Monascus* (*M. ruber, M. purpureus, M. pilosus,* and *M.floridanus*) [1]. In East Asia, RYR has been used to make red colored liquor and other fermented food products for more than 600 years [2]. RYR has been allowed as a food additive for the coloring of meat, fish, and soybean products in China since 1982 [3]. In Japan, pigments of *M. purpureus* are approved for foodstuffs [4]. Furthermore, it is authorized as a Health Functional Food for lowering serum cholesterol levels in Korea [5].

The major monacolin in RYR is monacolin K, which has an identical structure to lovastatin [6]. Lovastatin has been shown to inhibit the activity of HMG-CoA reductase, which is a key rate-limiting enzyme involved in cholesterol synthesis. Lovastatin lowers the levels of plasma cholesterol by inhibiting cholesterol synthesis and inducting LDL receptors in the liver [7]. In addition, RYR also contains unsaturated fatty acids, which may help reduce triglyceride levels [8]. Several intervention studies have shown that RYR lowers elevated serum cholesterol, LDL-C and triglyceride levels [9–13].

However, it is important to note that since RYR contains lovastatin, the adverse effects of RYR are potentially the same as for lovastatin in general [13]. Case reports of four sources (the World Health Organization (WHO), the French Agency for Food, Environmental and Occupational Health and Safety (ANSES), the Italian Surveillance system, and the Food and Drug Administration (FDA)) have described adverse effects of RYR (e.g., myopathy, rhabdomyolysis, liver function alteration, nervous system disorders, gastrointestinal disorders, and skin disorders) as seen in lovastatin treatment [14]. In particular, statins, including lovastatin, have been associated with muscle-related adverse events such as myopathy and rhabdomyolysis [15]. Myopathy and rhabdomyolysis are also the most reported adverse effects of RYR [14]. Muscular pain was observed with or without creatine phosphokinase (CPK) increase as an adverse effect of RYR supplements in case reports [16–18]. The next most important target of adverse events of RYR after the muscle is the liver [14]. There have been reports of people suffering from acute hepatitis and elevated alanine aminotransferase (ALT), aspartate aminotransferase (AST), and gamma-glutamyl transferase (GGT) levels after consuming RYR-based products [17–19]. The Italian Surveillance system reported cases of gastrointestinal disorders associated with RYR-related products, and these disorders consisted mostly of dyspepsia, nausea, vomiting and abdominal pain, and sometimes diarrhea [17]. In addition, the eruption of limbs, wheals, and pruritus cutaneous have been reported as adverse effects of food supplements containing RYR [17]. Also, some species of *M. purpureus* produce the mycotoxin citrinin, which is nephrotoxic [20]. In Korea, to ensure the safe consumption of RYR, the standard for citrinin in RYR as a health supplement ingredient is set at a maximum of 0.050 mg/kg, and this is regulated [5]. However, no regulations have yet been established for food ingredients in Korea. The European Commission Regulation No. 2023/915 sets the maximum permissible level of citrinin in rice fermented with *M. purpureus* at 0.1 mg/kg, considering the uncertainty surrounding its nephrotoxicity, carcinogenicity, and genotoxicity [21]. In April 2024, a fatal incident related to the consumption of RYR health products in Japan initially led to speculation that mold contamination and citrinin might be the cause. However, fortunately, following the Japanese Ministry of Health, Labour and Welfare’s announcement of the accident investigation in September, it was determined that the actual cause was the contamination of the product, puberulic acid, by improperly managed blue mold [22].

The European Food Safety Authority (EFSA) Panel concluded that monacolins from RYR were a significant safety concern at an intake level of 10 mg/day from the available information on the adverse effects reported in humans. Furthermore, the Panel considered that there were severe adverse effects of monacolin K from RYR at use levels as low as 3 mg/day [14]. According to the case reports related to RYR intake, there were severe adverse effects of monacolin K from RYR at use levels of 3–30 mg/day [16–19]. In eleven European countries, including France, Austria, and Italy, the recommended daily intake of monacolin K for food supplements containing RYR ranges from 2 to 48 mg/day according to the Mintel Global New Products Database (Mintel GNPD) [14]. In South Korea, the specification of Health Functional Food requires that RYR contain monacolin K at a daily intake level of 4–8 mg/day [5]. Since RYR is consumed as a food ingredient as well as a Health Functional Food in Korea, it can be postulated that the consumption level of monacolin K may exceed the recommended daily intake level set for Health Functional Foods. The chronic intake levels of RYR-based products in the aforementioned European countries and Korea overlap with the ranges of doses that cause severe adverse effects, so it is necessary to check the safety of RYR by carrying out a 90-day feeding study. However, there are few in vivo studies assessing the safety of RYR, and among them, there is no toxicity test that analyzes muscles, the most representative adverse effect target of RYR. This study has a novelty different from previous studies in terms of analyzing biochemical and histopathological parameters of the brain, digestive, respiratory, circulatory, reproductive, urinary and endocrine organs as well as muscle. And in the present study, we have checked the safety of RYR by carrying out a 90-day feeding study in male SD rats. There was a report in which RYR extract (22 mg monacolin K/day) was administered to male and female rats for 26 weeks, but the adverse effects only appeared in male rats; the absolute and relative weight of the liver increased with dose-dependence [23]. In addition, the main target organs (muscle, liver, nervous system, gastrointestinal system, and skin) for the adverse effects of monacolin K from RYR in humans are not sex-specific [14]. Therefore, in accordance with the 3Rs principle, we used only male rats in this study.

## Materials and methods

### Chemicals

Lovastatin (analytical standard) were obtained Sigma-Aldrich (St. Louis, MO, USA) and Citrinin (analytical standard) was purchased from Fujifilm Wako Pure Chemical Corporation (Osaka, Japan). A methanol solution of citrinin (10 mg/mL) was prepared as a stock and diluted with methyl alcohol to prepare working standards with required concentrations. All stock solutions were stored at – 20 ℃ prior to use. For HPLC analysis, solvents with a purity of at least 99.9% HPLC grade were used, while solvents for proximate analysis were of Guaranteed Reagent grade or higher.

### Test material

The RYR used in this experiment was prepared by fermenting white rice (*Oryza sativa*) with a 5% (v/w) inoculum of *Monascus pilosus***.** For the control group, white rice sourced from Ganghwa-gun, which was identical to the rice used for RYR production, was utilized. The products were purchased from a local market in Korea. All samples were stored at – 80 ℃ immediately after purchase, freeze-dried, and subsequently analysed for monacolin K and citrinin content using HPLC.

### Test diet

Test diets were prepared by replacing the corn starch of the AIN 93G with RYR and/or white rice. For the control diet, the whole corn starch of AIN 93G was replaced by white rice. The diet was replaced by a 1:1 mixture of RYR and white rice for the low-dose group and by RYR alone for the high-dose group. Therefore, the rats were fed with diets containing 0%, 20%, and 40% RYR, respectively. The compositions of the diets are shown in Table 1. All diets stored at 4  ℃ prior to use. And dietary nutrition, stability, and citrinin concentration of diet were confirmed analytically, as described later.

**Table 1:** The composition of test feeds

Ingredient (g/kg)	Group		
	Control	20% RYR	40% RYR
Casein	200.0	200.0	200.0
l-Cystine	3.0	3.0	3.0
White rice	397.5	198.7	0.0
Red yeast rice	0.0	198.7	397.5
Maltodextrin	132.0	132.0	132.0
Sucrose	100.0	100.0	100.0
Soybean oil	70.0	70.0	70.0
Cellulose	50.0	50.0	50.0
Mineral Mix, AIN-93G-MX (94046)	35.0	35.0	35.0
Vitamin Mix, AIN-93-VX (94047)	10.0	10.0	10.0
Choline Bitartrate	2.5	2.5	2.5
TBHQ, antioxidant	0.014	0.014	0.014

### Proximate analysis

The moisture and ash contents (gravimetric) were determined based on methods outlined by the Association of Official Agricultural Chemists (AOAC) [24]. The auto-Kjeldahl method was used to determine the crude protein content. The crude lipid was extracted with dimethyl ether using the Soxhlet method [24]. The crude fiber was determined by AOAC 978.10 [24]. The total carbohydrate content (%) was obtained by subtracting all other components from 100% (calculated as 100-(moisture + ash + crude protein + crude lipid + crude fiber)).

### Stability

Monacolin K, the marker compound of RYR, was quantified to verify the stability of the RYR contained in the feed [5]. The feed was stored at 4 ℃ throughout the experiment. The analytical samples were taken at the start and end of the feeding experiment. The experimental feed (0.5 g) was extracted with 10 ml of 75% ethanol using a sonicator (Ultrasonicator, Wisd, Gangwon, Korea) for 1 h at 60 ℃. The extract was centrifuged at 3000 rpm for 10 min at 25 ℃. The supernatant was filtered with a 0.2 μm pore size filter into a 1 ml vial, and monacolin K was quantified by HPLC coupled with a UV–Vis Detector (Ultimate 3000, Thermo Fisher Scientific, Waltham, MA, USA). The analytical column was a C18 HPLC column (VDSpher OptiAqua C18, 5 μm, 4.6 × 250 mm, VDS optilab, Berlin, Germany). The elution conditions involved a gradient of binary mobile phases: solvent A (phosphoric acid/water, 2/998, v/v) and B (acetonitrile). The gradient elution program was as follows: 0–30 min: solvent A from 65–35%, solvent B from 35–65%; 30–31 min: solvent A from 35–0%, solvent B from 65–100%, then holding for 14 min; 45–46 min: solvent A from 0–65%, solvent B from 100–35%, then holding until 60 min. The mobile-phase flow rate was 1.00 mL/min, and the oven temperature was 40 ℃. The chromatogram was detected by UV at 237 nm.

### Citrinin estimation in diet

The test feed (2.5 g) was extracted with 25 ml of 70% ethanol by shaking it with a rotating shaker (SI-600R, Lab Companion, Jeiotech, Seoul, Korea) at 200 rpm for 1 h at 40 ℃. The extract was centrifuged with 3000 rpm for 5 min at 25 ℃. The supernatant was collected and concentrated under reduced pressure (Multivapor P-12, Buchi, Flawil, Switzerland) at 50 ℃. The concentrated sample was dissolved in 1 ml of methanol. Subsequently, the solution was filtered with a 0.2 μm pore size filter into a 1 ml vial, and citrinin was analyzed by high performance liquid chromatography (HPLC; Ultimate 3000, Thermo Fisher Scientific, Waltham, MA, USA) coupled with a fluorescence detector (FLD; Ultimate 3000, Thermo Fisher Scientific, Waltham, MA, USA). The analytical column was a C18 HPLC column (VDSpher OptiAqua C18, 5 μm, 4.6 × 250 mm, VDS optilab, Berlin, Germany). The elution conditions involved a gradient of binary mobile phases: solvent A (phosphoric acid/water, pH was adjusted to 2.25) and B (acetonitrile). The gradient elution program was as follows: 0–9.45 min: solvent A from 50–20%, solvent B from 50–80%; 9.45–9.50 min: solvent A from 20–0%, solvent B from 80–100%, then holding for 10.5 min; 20–20.1 min: solvent A from 0–50%, solvent B from 100–50%, then holding until 30 min. The mobile-phase flow rate was 1.00 mL/min, and the oven temperature was 20 ℃. The excitation and emission wavelength were set at 331 nm and 500 nm, respectively.

### Animals and maintenance

Male Sprague–Dawley rats (five weeks old) were obtained from Dooyeol Biotech Co., Ltd. (Seoul, Korea). The experiment was conducted in accordance with ethical guidelines approved by the Institutional Animal Care and Use Committee (IACUC) of Seoul National University (approval number SNU-200721–3). The animals were housed in a room maintained at 22 ± 2 ºC with a relative humidity of 60–70% and exposed to a light and dark cycle of 12 h duration. During the experimental periods, drinking water and feed were provided ad libitum.

### Sub-chronic toxicity study

Rats were randomly divided into three groups: Control (0% RYR; n = 5), low-dose (20% RYR; n = 5); and high-dose (40% RYR; n = 5). Feed and water were given for 90 days. Food intake and body weight were measured weekly, and all animals were closely monitored for any clinical signs. At the end of the experimental period, animals were euthanized by cardiac puncture under isoflurane anesthesia and blood sample was collected. In addition, Organ weight measurements and gross pathological and histopathological examinations were conducted.

### Clinical chemistry

Blood was allowed to stand at room temperature for 30 min and then was centrifuged with 3000 rpm for 20 min at 4 ℃ to obtain the serum. The serum was stored at – 20 ℃ until analysis. A chemistry analyzer (DRI-CHEM 3500 s, FUJI Photo Film Co, Tokyo, Japan) was used to measure clinical parameters for GGT, ALT, AST, alkaline phosphatase (ALP), glucose (GLU), blood urea nitrogen (BUN), creatinine, total protein (TP), albumin (ALB), total bilirubin (T-BIL), calcium (Ca), inorganic phosphorus (IP), creatinine phosphokinase (CPK), triglyceride (TG), total cholesterol (T-CHOL), high-density lipoprotein cholesterol (HDL-C), and lactate dehydrogenase (LDH). Clinical parameters for sodium (Na), potassium (K), chloride (Cl), low-density lipoprotein cholesterol (LDL-C), thyroid stimulating hormone (TSH), thyroxine (T4), and triiodothyronine (T3) were commissioned to the Dooyeol Biotech (Seoul, Korea) for a clinical chemistry test.

### Organ weight and histopathological studies

The brain, heart, prostate, gastrocnemius, adrenal, thymus, spleen, pancreas, testis, epididymis, urinary bladder, seminal vesicle, liver, lungs, kidney, stomach, and thyroid were removed and weighed. The ratio of organ to brain weight was calculated. For all animals in the control and high-dose groups, the liver, lungs, kidney, brain, stomach, thyroid, urinary bladder, and gastrocnemius tissues were fixed in 10% formalin, and the fixed tissues were commissioned to the Wonkwang University of Health for a histopathology test. They were embedded in paraffin, stained with hematoxylin and eosin (H&E), and observed for any histological changes. A histopathological examination was also performed in one rat of the low-dose group for an epididymis in which visual findings were observed during autopsy.

### Statistical analysis

Body weight, organ weight (absolute and relative), and clinical chemistry parameters were compared via one-way ANOVAs. When an ANOVA yielded a significant result (*p* < 0.05), Dunnett’s t-test was used for comparisons between each treatment group and the control group. No further testing was performed for non-significant ANOVA results. Pearson's correlation coefficient (r) was used to determine the correlation between RYR contents and food or energy efficiency.

## Results

### Feed composition

The proximate analysis of the experimental feeds revealed no significant differences in their moisture, carbohydrate, protein, ash, and fiber. However, the feeds of the low- and high-dose groups had significantly higher fat (20.9% and 29.8%, respectively) and energy (3.05% and 3.33%, respectively) than those of the control group (Table 2).

**Table 2:** Proximate composition of the experimental feeds

Group	Control	20% RYR	40% RYR
g/100 g diet			
Moisture	11.45 ± 0.37	10.12 ± 0.94	10.27 ± 0.62
Carbohydrates	56.83 ± 0.49	57.80 ± 2.63	56.37 ± 0.64
Protein	21.13 ± 0.12	20.40 ± 1.85	21.03 ± 0.99
Fat	5.37 ± 0.45	6.49 ± 0.20*	6.97 ± 0.22*
Ash	3.97 ± 0.03	3.91 ± 0.07	4.01 ± 0.09
Fiber	1.25 ± 0.65	1.26 ± 0.21	1.34 ± 0.50
kcal/100 g diet			
Energy	360.3 ± 3.5	371.3 ± 4.7*	372.3 ± 0.6**

Values are mean ± SD (n = 3)

Significantly different from control by Dunnett’s t-test: *p < 0.05, **p < 0.01

### Stability test of feeds

The result of stability test is presented in Table 3. There was no significant difference in monacolin K content between day 0 and day 90 for both the low-dose and high-dose feeds.

**Table 3:** Monacolin K content in the experimental feeds (comparison between day 0 and day 90)

Group	μg Monacolin K/g diet	
	Day 0	Day 90
20% RYR	23.9 ± 1.2	24.3 ± 2.1
40% RYR	47.0 ± 3.6	49.3 ± 2.9

No significant difference in monacolin K content between day 0 and day 90 for both the low-dose and high-dose feeds (*p* < 0.05)

### Citrinin contents in feeds

The LOD values were determined by multiplying the ratio of the standard deviation to the slope by 3.3 times. The LOD value of the citrinin was 116.18 ppb. In the analysis of the experimental feeds, all values were below LOD.

### Clinical signs and mortality

There were no clinical signs of toxicity or mortality during the experimental period at any of the dosage levels.

### Body weight gain, food intake, and food efficiency

**Fig. 1:**
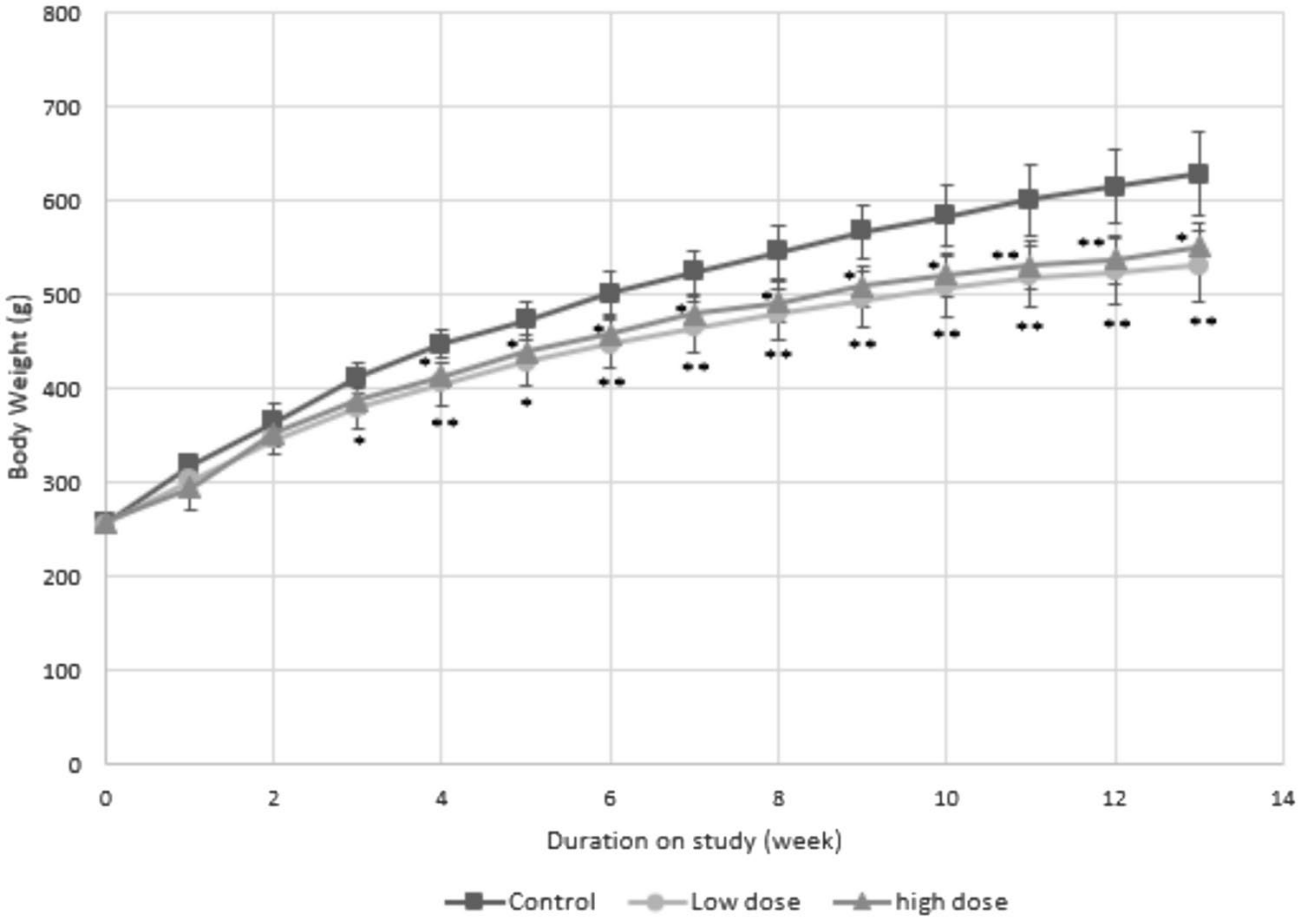
The changes in body weight of male SD rats fed with RYR for 90 days. Each data point is presented as mean ± standard deviation. *p < 0.05 and **p < 0.01 are considered significantly different from the control group values

During the study period, the low and high-dose groups experienced a significant reduction (*p* < 0.01) in total weight gains (– 25.7% and – 20.9%, respectively) compared to that of the control group (Fig. 1, Table 4). The average food intake per cage was used as the food intake. The food efficiency and energy efficiency were calculated by dividing the average weight gain per cage by the food intake and by dividing the average weight gain per cage by the energy intake (Table 4). A decrease in food efficiency (– 14.95% and – 15.49% for the low and high dose, respectively) as well as energy efficiency (– 17.32% and – 18.18% for the low and high dose, respectively) compared to the control group was observed in both experimental groups (Table 4).

**Table 4:** The food efficiency and intake of food, energy, RYR, and monacolin K of male SD rats fed with RYR for 90 days

Group	Control	20% RYR	40% RYR
Total weight gain(g)	370.6 ± 40.1	275.4 ± 30.9**	293.0 ± 15.9**
Average weight gain(g/day)	4.12 ± 0.45	3.06 ± 0.34**	3.26 ± 0.18**
Average food intake(g/day)	24.97 ± 0.49	21.20 ± 1.29	23.40 ± 2.52
FE^a^	0.1666 ± 0.0096	0.1417 ± 0.0080	0.1408 ± 0.0064
Average energy intake(kcal/day)	89.98 ± 1.76	78.73 ± 4.80	87.11 ± 9.37
EE^b^	0.0462 ± 0.0027	0.0382 ± 0.0022	0.0378 ± 0.0017
Red yeast rice intake(g/kg b.w./day)	0	10.82 ± 0.16	22.84 ± 1.49
Monacolin K intake(mg/kg b.w./day)	0	1.310 ± 0.020	2.767 ± 0.180

Values are mean ± SD (n = 5)

^a^FE (food efficiency) = Average weight gain (g/day)/Average food intake (g/day)

^b^EE (energy efficiency) = Average weight gain (g/day)/Average energy intake (kcal/day)

### Clinical chemistry

Except for the ALT, there were no significant differences in the clinical chemistry parameters in the low- and high-dose rats compared to those of the control rats (Table 5). The ALT of the low- and high-dose groups decreased compared with that of the control group (*p* < 0.05). However, all clinical chemistry parameters, excepting T4 (thyroxine), fell within the range of reference data of SD rats [25–31]. There was an increase in T4 levels compared to the control group in the low- and high-dose groups, without statistical significance (Table 5).

**Table 5:** The clinical chemistry of male SD rats fed with RYR for 90 days

Parameter	Unit	Group		
		Control	20% RYR	40% RYR
GGT	(U/l)	< 1	< 1	< 1
ALT	(U/l)	23.4 ± 4.3	16.6 ± 2.1*	18.2 ± 2.8*
AST	(U/l)	92.2 ± 29.2	66.2 ± 16.3	60 ± 5.0
ALP	(U/l)	360.4 ± 73.7	317.4 ± 73.3	305.4 ± 64.7
GLU	(mg/dl)	120 ± 22.6	132.4 ± 34.8	103 ± 19.3
BUN	(mg/dl)	12.7 ± 1.9	15.5 ± 2.8	14.2 ± 1.2
CREATININE	(mg/dl)	0.272 ± 0.037	0.322 ± 0.053	0.284 ± 0.035
TP	(g/dl)	6.56 ± 0.21	6.06 ± 0.38	6.00 ± 0.47
ALB	(g/dl)	3.90 ± 0.24	3.62 ± 0.40	3.64 ± 0.34
T-BIL	(mg/dl)	0.66 ± 0.45	0.36 ± 0.05	0.54 ± 0.43
Ca	(mg/dl)	10.46 ± 0.43	10.30 ± 0.93	10.14 ± 0.40
IP	(mg/dl)	8.20 ± 0.95	8.16 ± 1.01	7.84 ± 0.59
CPK	(U/l)	443.0 ± 221.8	284.4 ± 108.3	231.6 ± 61.6
Na	(mmol/L)	145.8 ± 3.7	150.2 ± 8.9	150.4 ± 3.8
K	(mmol/L)	6.66 ± 0.71	6.28 ± 1.21	7.54 ± 0.82
Cl	(mmol/L)	103.0 ± 0.7	105.4 ± 3.4	106.4 ± 0.9
TG	(mg/dl)	81.4 ± 40.0	76.4 ± 20.6	91.2 ± 30.6
T-CHOL	(mg/dl)	83.0 ± 15.7	80.2 ± 10.5	72.8 ± 7.1
LDL-C	(mg/dl)	7.54 ± 0.84	6.90 ± 1.41	6.88 ± 1.01
HDL-C	(mg/dl)	52.6 ± 10.5	49.0 ± 4.5	46.2 ± 5.2
LDH	(U/l)	810.6 ± 484.9	551.2 ± 324.0	547.0 ± 229.4
TSH	(ng/mL)	2.68 ± 2.10	1.58 ± 0.92	1.80 ± 1.03
T4	(ng/mL)	50.49 ± 14.61	76.17 ± 12.55	75.73 ± 51.64
T3	(ng/mL)	1.54 ± 0.27	1.61 ± 0.05	1.57 ± 0.02

*GGT* gamma-glutamyltransferase, *ALT* alanine aminotransferase, *AST* aspartate aminotransferase, *ALP* alkaline phosphatase *GLU* glucose, *BUN* blood urea nitrogen, *TP* total protein, *ALB* albumin, *T-BIL* total bilirubin, *Ca* calcium, *IP* inorganic phosphorus, *CPK* creatinine phosphokinase, *Na* sodium, *K* potassium, *Cl* chloride, *TG* triglyceride, *T-CHOL* total cholesterol, *LDL-C* low-density lipoprotein cholesterol, *HDL-C* high-density lipoprotein cholesterol, *LDH* lactate dehydrogenase, *TSH* thyroid stimulating hormone, *T4* thyroxine, *T3* triiodothyronine

All data are given as mean ± standard deviation (n = 5)

*Significantly different from control by Dunnett’s t-test at *p* < 0.05

### Organ weights

The absolute organ weights and the ratios of organ to brain weight in all groups are shown in Table 6. There were significant decreases in the liver, lungs, kidney, stomach, and thyroid weight in the low-dose group compared to those of the control group (*p* < 0.01, *p* < 0.05, *p* < 0.01, *p* < 0.01, and *p* < 0.05, respectively). Among them, the relative weights of liver, kidney, and stomach to brain in the low-dose group showed a significant decrease compared to those of the control group (*p* < 0.01, *p* < 0.05, and *p* < 0.05, respectively). There was no significant difference in the absolute or relative organ weights in the high-dose group compared to those of the control group.

**Table 6:** The absolute organ weights (g) and the relative organ weights (g/g brain weight) of male SD rats fed with RYR for 90 days

	Absolute organ weights			Relative organ weights		
	Control	20% RYR	40% RYR	Control	20% RYR	40% RYR
Brain	2.25 ± 0.19	2.06 ± 0.05	2.16 ± 0.10	1.00 ± 0.00	1.00 ± 0.00	1.00 ± 0.00
Heart	1.65 ± 0.15	1.56 ± 0.11	1.54 ± 0.06	0.73 ± 0.06	0.76 ± 0.06	0.72 ± 0.05
Prostate	1.50 ± 0.40	1.75 ± 0.10	1.58 ± 0.20	0.67 ± 0.16	0.85 ± 0.07	0.73 ± 0.08
Gastrocnemius	4.35 ± 0.36	4.11 ± 0.24	4.27 ± 0.22	1.93 ± 0.03	2.00 ± 0.12	1.98 ± 0.14
Adrenal	0.0698 ± 0.0090	0.0734 ± 0.0060	0.0750 ± 0.0080	0.0319 ± 0.0043	0.0356 ± 0.0029	0.0349 ± 0.0044
Thymus	0.55 ± 0.13	0.42 ± 0.08	0.44 ± 0.07	0.2501 ± 0.0802	0.2062 ± 0.0381	0.2038 ± 0.0251
Spleen	1.04 ± 0.21	0.97 ± 0.20	0.88 ± 0.10	0.46 ± 0.10	0.47 ± 0.10	0.41 ± 0.03
Pancreas	1.65 ± 0.44	1.95 ± 0.48	1.68 ± 0.32	0.73 ± 0.16	0.95 ± 0.24	0.78 ± 0.15
Testis	3.95 ± 0.37	4.09 ± 0.24	3.67 ± 0.72	1.76 ± 0.24	1.99 ± 0.15	1.70 ± 0.30
Epididymis	1.58 ± 0.24	1.68 ± 0.34	1.31 ± 0.35	0.71 ± 0.11	0.82 ± 0.16	0.60 ± 0.15
Urinary bladder	0.13 ± 0.02	0.12 ± 0.02	0.15 ± 0.02	0.0591 ± 0.0122	0.0559 ± 0.0075	0.0689 ± 0.0111
Seminal vesicle	1.95 ± 0.22	1.92 ± 0.35	1.65 ± 0.22	0.87 ± 0.07	0.94 ± 0.18	0.76 ± 0.08
Liver	16.47 ± 1.70	12.56 ± 1.18**	14.64 ± 0.73	7.32 ± 0.49	6.10 ± 0.54**	6.80 ± 0.45
Lungs	2.07 ± 0.26	1.74 ± 0.13*	1.88 ± 0.14	0.93 ± 0.15	0.85 ± 0.08	0.87 ± 0.04
Kidney	4.02 ± 0.34	3.15 ± 0.28**	3.61 ± 0.22	1.79 ± 0.07	1.53 ± 0.13*	1.67 ± 0.07
Stomach	2.21 ± 0.14	1.78 ± 0.13**	2.11 ± 0.06	0.98 ± 0.06	0.87 ± 0.07*	0.98 ± 0.07
Thyroid	0.0550 ± 0.0087	0.0410 ± 0.0098*	0.0504 ± 0.0033	0.0247 ± 0.0051	0.0199 ± 0.0049	0.0234 ± 0.0022

All data are given as mean ± standard deviation (n = 5)

Significantly different from control by Dunnett’s t-test: *p < 0.05, **p < 0.01

### Histopathology

There were no abnormal histopathological findings in the brain, urinary bladder, or gastrocnemius of the control and high-dose groups (Table 7). We focused on the histopathological findings shown only in the high-dose groups. A focal foamy macrophage infiltrate was observed in one object in the high-dose group, but this can be observed in normal lungs [32]. A case of basophilic cortical tubules was observed in the high-dose group, but it is also considered a normal feature in young growing rats, and it can be increased in an age-dependent manner [33]. In addition, the hyperplasia and hypertrophy in thyroids were observed in two objects in the high-dose group and are expected as a systemic response to increased pituitary secretion of TSH [34]. However, thyroid hormone tests showed no increase in TSH levels in the low- or high-dose group. In summary, except for the hyperplasia and hypertrophy in the thyroid, since all the histopathological observations were events that have often been reported in normal animals, they were considered to not be toxicologically significant.

**Table 7:** The summary of histopathological findings

Organ		Group (No. of Animals)	
		Control	40% RYR
	Examined	5	5
Brain			
	No finding noted	5	5
Urinary bladder			
	No finding noted	5	5
Gastrocnemius			
	No finding noted	5	5
Liver			
focal steatosis	Number of affected	4	0
Stomach			
Focal glandular dilatation	Number of affected	1	0
Lung			
Alveolar wall thickening	Number of affected	5	5
Alveolar congestion & edema	Number of affected	5	5
Focal peribronchial inflammatory cell infiltrates	Number of affected	1	0
Focal foamy macrophages infiltrates	Number of affected	0	1
Kidney			
A few atrophy glomeruli	Number of affected	5	5
Protein casts	Number of affected	5	4
Focal interstitial inflammatory cell infiltrates	Number of affected	2	2
Cystic dilatation of tubules	Number of affected	2	2
Focal tubular degeneration	Number of affected	1	0
Focal areas of tubule basophilia	Number of affected	0	1
Thyroid			
Minimal follicular cell hypertrophy & hyperplasia	Number of affected	0	2

## Discussion

We conducted a 90-day feeding study to check the safety of RYR as a general food ingredient. The daily intake of RYR and monacolin K in the low-dose group was 10.82 g/kg b.w./day and 1.310 mg/kg b.w./day, and in the high-dose group, was 22.84 g/kg b.w./day and 2.767 mg/kg b.w./day, respectively. Rats treated with RYR showed lower body weight gain as accompanied by low food efficiency. There were no signs of toxicity in serum clinical chemistry or relative organ weights, while the hyperplasia and hypertrophy in thyroids were observed in two objects in the high-dose group, and the low- and high-dose groups showed increases in T4 levels.

Citrinin is a mycotoxin produced by certain species of *M. purpureus* [20]. Its regulation varies internationally, with different countries setting distinct permissible limits for citrinin content in fermented red rice products. The European Union (EU) has established the maximum allowable level of 100 μg/kg in food supplements based on rice fermented with *M. purpureus* [21], while Japan and China set their limits at 200 μg/kg and 50 μg/kg in fermented red rice, respectively [35]. Taiwan allows up to 2000 μg/kg [36], and South Korea has no specific legal limits for citrinin in general food products; however, for functional foods, it is regulated at a maximum of 50 μg/kg [5]. In the present study, the citrinin concentration in all feed samples was below the detection limit of 116.18 μg/kg, which is within the regulatory thresholds established by the aforementioned countries. Additionally, the estimated citrinin intake was less than 2.79 μg/kg b.w./day, which is below the NOAEL of 20 μg/kg b.w./day, as reported in a previous 90-day oral toxicity study in rats[37]. Based on these findings, the contribution of citrinin to any observed toxicological effects in this study is considered negligible.

Although RYR is widely recognized for its lipid-lowering effects, it contains monacolin K, a naturally occurring HMG-CoA reductase inhibitor, and thus has been associated with adverse effects similar to those of lovastatin. These include myopathy, rhabdomyolysis, liver dysfunction, gastrointestinal disturbances, and dermatological reactions [13, 14]. Among these, muscle-related side effects, particularly myopathy and rhabdomyolysis, have been the most frequently reported in human case studies [14, 15]. Liver-related adverse effects, including acute hepatitis and elevated liver enzymes (AST, ALT, GGT), have also been documented [17–19]. In the present study, however, no histopathological abnormalities were observed in the muscle (gastrocnemius) or liver tissues of either the control or high-dose groups (Table 7). Although serum ALT levels were significantly decreased in both the low- and high-dose groups compared to the control (*p* < 0.05), all other serum biochemical parameters remained within the normal reference ranges[25–31]. Importantly, a reduction in ALT alone is generally not considered toxicologically significant[38]. While reductions in absolute and relative liver weights were observed in the low-dose group, these changes were not dose-dependent and were not accompanied by biochemical or histopathological alterations. Therefore, these findings are interpreted as non-adverse effects possibly related to individual variation or feed intake, rather than RYR-induced organ toxicity.

The low- and high-dose groups experienced a significant reduction in total weight gain (– 25.7% and – 20.9%, respectively; *p* < 0.01) compared to the control group. Decrements in body weight gain by more than 10% compared to the control group are considered as a sensitive indicators of toxicity [39]. In addition, a decrease in food efficiency was observed in the low- and high-dose groups compared to the control group. As a result of Pearson correlation analysis, the Pearson correlation coefficient (r) between the RYR content and food efficiency was – 0.628 (*p* < 0.05) and that between the RYR content and energy efficiency was – 0.706 (*p* < 0.01), which shows dose-dependency. These results suggest that RYR might inhibit growth in rats. The growth inhibition effect of RYR could partially be associated with the inhibition of the growth of *Metanobrevibacter smithii,* which lives in the intestine of rats. *M. smithii* is one of the methane-producing microbes that generates methane in the gut by utilizing hydrogen and carbon dioxide. During this process, undigested polysaccharides are fermented by gut bacteria into short-chain fatty acids (SCFAs), which are absorbed in the gut and used as an energy source in the body. In other words, *M. smithii* has a significant impact on the host's energy extraction efficiency. Faseleh Jahromi, et al*.* reported that HMG-CoA reductase inhibitors (such as lovastatin) inhibit the growth of *M. smithii* [34]. Additionally, a study by Samuel and Gordon showed that when *Bacteroides thetaiotaomicron* and *M. smithii* were co-colonized in mice, there was a greater weight gain compared to the colonization of *B. thetaiotaomicron* alone [40]. Notably, *M. smithii* constitutes approximately 94% of the methane-producing microbial population in the human digestive tract [41]. Therefore, the intake of RYR may influence human nutritional efficiency. Further, RYR also contains various sterols such as β-sitosterol, campesterol, stigmasterol, and sapogenin [2, 10, 11]. Phytosterols have also been reported to be involved in the reduction of weight gain and are known to directly affect intestinal energy absorption [40, 42].

The ratio of organ weight to brain weight can be a more reliable indicator of toxicity than the ratio of organ weight to body weight; this is because brain weight is rarely affected nonspecifically by toxicity, whereas body weight is more variable and may change as a result of toxicity [39]. There were significant differences in organ to brain weight only in the low-dose group compared to the control group. Since no dose-dependency nor changes in other markers (biochemical or histopathological) for corresponding organs were observed, it was difficult to verify organ toxicity.

RYR is known to lower the serum concentration of total cholesterol, LDL-C, and triglyceride. In the present study, lipid lowering effect was not observed. There were slight reductions in total cholesterol and LDL-C levels in the low- and high-dose rats compared to the control rats, without statistical significance and no decrease in triglyceride level. These results are concerned to be because this study focused on adverse effects and, unlike other efficacy studies, was conducted on normal rats that were not hyperlipidemic.

The decrease in body weight gain and the histopathological alterations observed in thyroid tissue in the high-dose group (refer to Results) prompted an investigation of thyroid hormone levels. Although not statistically significant, the low- and high-dose groups exhibited increases in serum T4 levels (50.89% and 49.99%, respectively). These trends suggest a potential association between RYR intake and altered thyroid function. Furthermore, the follicular cell hyperplasia and hypertrophy observed in the high-dose group are reminiscent of changes previously reported in rats treated with simvastatin, another HMG-CoA reductase inhibitor [43], suggesting a potential role for monacolin K. In the study by Smith et al*.*, simvastatin treatment induced hepatomegaly and thyroid hypertrophy, which were associated with increased hepatic functional capacity and enhanced thyroxine clearance, ultimately resulting in elevated TSH levels [43]. The TSH elevation was presumed to mediate the observed proliferation and hypertrophy of thyroid follicular cells [43]. However, in the present study, although the changes were not statistically significant, a trend toward increased serum T4 levels was observed, making it difficult to directly apply the mechanistic conclusions of Smith et al*.*'s findings to our results. Additionally, no significant differences in TSH or T3 levels were found compared to the control group, suggesting that dysregulation of the pituitary–thyroid axis alone may not fully explain the observed thyroid histopathology. Alternatively, secondary factors such as metabolic stress or nutritional imbalance induced by the 90-day RYR administration may have contributed to these histological alterations. Weight gain loss and reduced caloric efficiency may have influenced systemic endocrine responses, including thyroid activity [44]. Taken together, these findings highlight the need for further investigations to elucidate the mechanisms by which RYR modulates thyroid follicular cell dynamics and to determine how these effects may be linked to the growth-inhibitory outcomes observed in RYR-treated rats.

In summary, the results of this study suggest that red yeast has subchronic toxic potential, particularly affecting weight gain, food efficiency, and thyroid function in both low and high-dose groups. Reductions in body weight gain are recognized as sensitive indicators of toxicity [39] and may have serious adverse effects during developmental periods such as childhood and adolescents. This study was conducted in SD rats aged 6 to 19 weeks, which corresponds to the human age range of approximately 6 to 14 years based on weaning and sexual maturity [45]. Therefore, our findings support that children and adolescents should be more cautious about RYR intake. In addition, further studies are warranted to clarify the mechanisms by which RYR influences growth and thyroid function and to determine safe intake levels, especially for children and adolescents.

## Data Availability

The data that support the findings of this study are available from the author upon reasonable request.
